# Oligosaccharides as Potential Therapeutics against Atherosclerosis

**DOI:** 10.3390/molecules28145452

**Published:** 2023-07-17

**Authors:** Siarhei A. Dabravolski, Vasily N. Sukhorukov, Alexandra A. Melnichenko, Victoria A. Khotina, Alexander N. Orekhov

**Affiliations:** 1 Department of Biotechnology Engineering, Braude Academic College of Engineering, Snunit 51, P.O. Box 78, Karmiel 2161002, Israel; 2 Institute of General Pathology and Pathophysiology, 8 Baltiyskaya Street, 125315 Moscow, Russia; vnsukhorukov@gmail.com (V.N.S.); sasha.melnichenko@gmail.com (A.A.M.); nafany905@gmail.com (V.A.K.); a.h.opexob@gmail.com (A.N.O.)

**Keywords:** atherosclerosis, chronic inflammation, oligosaccharide, hyperlipidaemia, oxidative stress

## Abstract

Atherosclerosis is the major cause of cardiovascular-disease-related death worldwide, resulting from the subendothelial accumulation of lipoprotein-derived cholesterol, ultimately leading to chronic inflammation and the formation of clinically significant atherosclerotic plaques. Oligosaccharides have been widely used in biomedical research and therapy, including tissue engineering, wound healing, and drug delivery. Moreover, oligosaccharides have been consumed by humans for centuries, and are cheap, and available in large amounts. Given the constantly increasing number of obesity, diabetes, and hyperlipidaemia cases, there is an urgent need for novel therapeutics that can economically and effectively slow the progression of atherosclerosis. In this review, we address the current state of knowledge in oligosaccharides research, and provide an update of the recent in vitro and in vivo experiments that precede clinical studies. The application of oligosaccharides could help to eliminate the residual risk after the application of other cholesterol-lowering medicines, and provide new therapeutic opportunities to reduce the associated burden of premature deaths because of atherosclerosis.

## 1. Introduction

Atherosclerosis is the major cause of various atherosclerotic cardiovascular diseases, such as peripheral artery disease, ischaemic stroke, and coronary artery disease. As a chronic inflammatory disease, atherosclerosis is characterised by a pathological lipid accumulation, and remodelling of the arterial wall. The outcome of atherosclerosis depends on the interplay between many genetic and environmental risk factors, with the degree of inflammation and hyperlipidaemia playing the main role in its pathogenesis [1]. In total, advanced atherogenesis and its complications cause 17.9 million deaths or 32% of all deaths per year worldwide [2].

Atherosclerosis results from an influx of cholesterol-rich low-density lipoprotein (LDL) particles, coated with apolipoprotein B (ApoB) protein, in the intimal layer of the arterial wall in atherosclerosis-susceptible regions of the arterial tree, such as in arterial bifurcations [3]. In the intimal layer, the LDL particles are subjected to various proteolytic and lipolytic enzymes, and oxidative agents, thus forming multiple modified low-density lipoprotein (mmLDL) particles. An MmLDL accumulation in the intima of blood vessels causes the dysfunction of endothelial cells (ECs), which is characterised by an increased expression of inflammatory mediators (such as interleukins (*IL-1β*, *IL-6*), *TNF-α*, and *interferon-gamma* (*IFN-γ*)) and adhesion molecules (such as *ICAM* (*Intercellular Adhesion Molecule-1*), *VCAM* (*Vascular Cell Adhesion Molecule*-*1*), and *P*- and *E*-*selectins*), and thus promotes inflammation and an increasing leukocyte adhesiveness. Further, macrophages uptake mmLDL and form foam cells, which facilitate inflammation, and stimulate humoral and adaptive immunity [4,5].

Oxidative stress is another essential player in the initiation and progression of endothelial dysfunction and atherosclerosis [6]. An increase in ROS (reactive oxygen species), and insufficient antioxidant production, facilitate lipid deposition, and promote inflammation and endothelial injury [7]. There are several other processes associated with the disease’s progression. The transition of epithelial cells to mesenchymal cells, and the differentiation of fibroblasts to myofibroblasts under the influence of inflammatory cytokines and pro-apoptotic regulators is known to reinforce endothelial dysfunction and plaque formation. Furthermore, the plaque at advanced stages could necrotise macrophages and smooth muscle cells, thus resulting in the formation of a plaque necrotic core, often covered by a fibrous cap, which is prone to rupture and cause thrombosis and vessel occlusion [8]. Additionally, the dysregulation of Ca^2+^ homeostasis in the plaque initiates a mineralisation process similar to bone formation, which leads to plaque calcification [9]. Extended calcifications are known to stabilise the plaque, while spotty calcifications increase the plaque’s vulnerability and chance of rupturing [10]. Therefore, an efficient anti-atherosclerotic treatment is usually based on lipid-lowering, antioxidant, and/or anti-inflammatory properties [11].

Oligosaccharides are formed by a small number (usually 3–10) of monosaccharide monomers, and are associated with various biological functions (cell adhesion and recognition, carbohydrate storage and transport, prebiotic effects). Oligosaccharides are normally linked to lipids or to amino acids via N- or O-glycosidic bonds, thus creating glycolipids or glycoproteins, respectively [12]. Other forms of oligosaccharides (not linked to lipids or amino acids) may be presented, for example, by the plant raffinose series, which perform the storage function, or maltodextrins and cellodextrins, which result from the microbial breakdown of larger polysaccharides, such as starch or cellulose [13]. In addition to the wide application of natural oligosaccharides (such as fructo-oligosaccharides (FOSs), galacto-oligosaccharides (GOSs), human milk oligosaccharides (HMOs), Mannan oligosaccharides (MOSs) and others) in human diets and animal feed [14], different synthetic analogues of natural oligosaccharides, created through a combination of modern chemical synthesis, computational, and analytical methods, have demonstrated high potential for applications in biology, nanotechnology, material science, and medicine [15].

Further on in this review, we focus on the recent studies investigating the molecular mechanisms of oligosaccharides and their derivatives used to treat atherosclerosis. The application of polysaccharides as therapeutic agents to treat atherosclerosis was recently reviewed [16,17] and, therefore, will be excluded from our review. Additionally, the oligosaccharide-gut microbiota axis has been extensively reviewed in several recent papers, towards which we wish to redirect interested readers [18,19,20].

## 2. Alginate Oligosaccharide

Alginate is a polysaccharide, the major component of the brown algae cell wall (approximately 40% of the dry weight), formed by the monomeric units of β-D-mannuronate and α-L-guluronate, linked through a β-1,4-glycosidic bond. Alginate oligosaccharide (AOS) is delivered via the de-polymerisation (for example, via acid hydrolysis, oxidation, and enzymatic degradation) of alginate, and is widely used in a variety of fields, including drug delivery and tissue engineering applications [21]. The unique advantageous properties (such as being water-soluble, biodegradable, non-toxic, and non-immunogenic) and activities (anti-inflammatory, anti-proliferative, antioxidative and anti-apoptotic) of AOS explain the growing interest it is receiving from the scientific community [22].

Recent in silico and in vitro investigations showed the high affinity of both polysaccharide forms (sulfated (fucoidan) and non-sulfated (alginate)) to L- and E-selectin, monocyte chemoattractant protein 1 (MCP-1), and ICAM-1, with no signs of cytotoxicity for THP-1 macrophages, even at high concentrations. The interaction with these inflammatory markers was manifested in vitro by an inhibited migration and reduced expression of MCP-1 and ICAM-1 in IFN-γ-induced THP-1 monocytes [23]. Experiments in vivo suggested that AOS protected mice against the cardiotoxic effects of the chemotherapeutic drug doxorubicin (DOX) (Table 1) [24], which is mediated through oxidative stress and endoplasmic reticulum (ER) apoptosis [25]. AOS application in mice greatly increased their survival rate, improved their DOX-induced cardiac dysfunction, and attenuated myocardial apoptosis. On the molecular level, AOS treatment decreased the expression of pro-apoptotic proteins (*C/EBP homologous protein* (*CHOP*) and *apoptosis regulator Bcl-2-associated X protein* (*Bax*)) and increased the expression of the anti-apoptotic protein *B-cell lymphoma-2* (*Bcl-2*) [24]. A similar mechanism was also involved in AOS-mediated protection against myocardial ischaemic/reperfusion injury in mice. AOS treatment effectively decreased the generation of superoxide, 4-hydroxynonenal, and 3-nitrotyrosine, and down-regulated NADPH oxidase 2 and inducible nitric oxide synthase, thus attenuating oxidative stress. The decrease of myocardial apoptosis was reflected by the up-regulation of the anti-apoptotic Blc-2 protein, and the down-regulation of pro-apoptotic factors (CHOP, caspase-12, Bax, and glucose-regulated protein 78). Finally, AOS application greatly decreased the infarct size, and ameliorated cardiac dysfunction, after I/R injury in mice [26].

Recent research on human umbilical vein endothelial cells (HUVECs) subjected to oxidative stress revealed that AOS regulated the expression of *P21*, *focal adhesion kinase* (*FAK*), *cyclin-dependent kinase 2* (*CDK2*), *integrin-α*, *phosphatase and tensin homolog* (*PTEN*), and *phosphoinositide 3-kinase alpha* (*PI3K*), suggesting the involvement of the integrin-α/FAK/PI3K pathway. Additionally, AOS treatment protected HUVECs against oxidative stress-induced apoptosis by increasing the expression of *Bcl-2*, and decreasing the levels of caspase 3 and Bax [27]. As has been established, the integrin-α/FAK/PI3K pathway is involved in the regulation of various cellular processes, such as the cell cycle, signal transduction, cell proliferation, adhesion, and others, thus making it an important therapeutic target [54,55,56]. In another study, AOS effectively protected HUVECs against H_2_O_2_-induced oxidative stress and apoptosis, by up-regulating antioxidant enzymes (superoxide dismutase (SOD) and catalase (CAT)) and glutathione, and reducing the activities of caspase-3 and caspase-9 [28].

Furthermore, AOS has been suggested as a therapeutic agent to alleviate cardiac ageing. In a mouse model of D-galactose-induced ageing, AOS administration preserved the ejection fraction and fractional shortening. On the molecular level, OAS inhibited the D-galactose-induced up-regulation of ageing markers p21 and p53, and the cardiac peptide hormones brain natriuretic peptide (BNP) and natriuretic peptide A (ANP). AOS treatment also increased the mtDNA copy number and autophagy rate, sustained the mitochondrial membrane potential, and up-regulated the expression of *peroxisome proliferator-activated receptor gamma coactivator 1-alpha* (*PGC-1α*) and *sirtuin-3* (*SIRT3*), thus improving mitochondrial biogenesis, turn-over, and integrity [29].

Together, these finding indicate that alginate is a potent compound which can protect endothelial cells against oxidative stress, endoplasmic reticulum stress-mediated apoptosis, and inflammation, thus providing novel alternative strategies to prevent atherosclerosis in the future. However, the efficiency of AOS has been demonstrated in a limited number of in vitro experiments and in vivo models. Therefore, future studies should focus on elucidating the exact molecular mechanisms regulating these beneficial effects, and the pharmacokinetics of AOS.

## 3. Chitosan Oligosaccharide

Chitosan oligosaccharide (COS) is known as an effective lipid-lowering natural material. COS’ unique biological activities and physico-chemical properties allow it to normalise levels of very-low-density lipoprotein (VLDL), LDL, and high-density lipoprotein (HDL), which is especially valuable in the treatment of diabetes mellitus, obesity, hyperlipidaemia, hyperglycaemia, metabolic syndrome, and associated diseases and morbidities [57,58].

**Figure 1:**
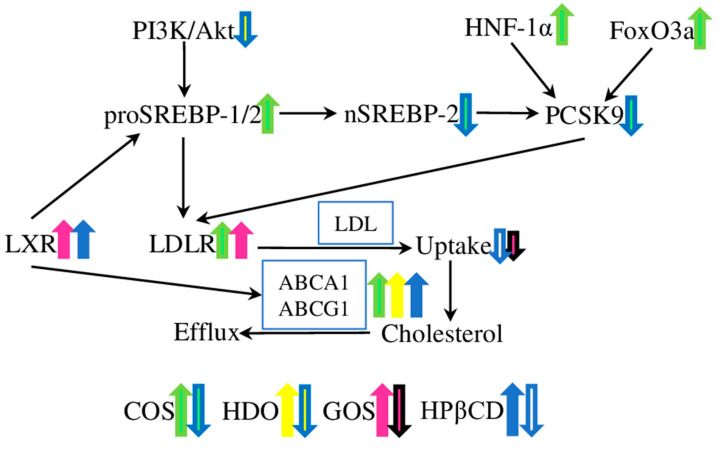
The effects of oligosaccharides on cholesterol and lipid metabolism. The low-density lipoprotein receptor (LDL-R), ATP-binding cassette transporters A1 and G1 (ABCA1 and ABCG1), and liver X receptor (LXR) are the key targets of the COS and GOS, COS, HDO and HPβCD, GOS and HpβCD, respectively. Chitosan oligosaccharide (COS) treatment increased the lipid intake by the liver through the regulation of three transcription factors; forkhead box O3 (FOXO3a), sterol-responsive element-binding protein 2 (SREBPs), and hepatocyte nuclear factor-1α (HNF-1α); which decreased the level of the LDL-R inhibitor proprotein convertase subtilisin/kexin type 9 (PCSK9). Heparin-derived oligosaccharides (HDO) down-regulated the protein kinase B/phosphoinositide 3-kinase (Akt/PI3K); one of the major regulators of cellular metabolism, growth, and proliferation; and up-regulated the ABCA1 transporter. Galacto-oligosaccharides (GOSs) and 2-hydroxypropyl-β-cyclodextrin (HPβCD) acted through LRX and ABCA1 and ABCG1 in an LDL-R-dependent way, decreasing the levels of triglycerides, LDL, and very-low-density lipoprotein (VLDL) cholesterol.

COS administration decreased the lesion and plaque areas in the aortic root, increased plaque stability, and reduced plasma triglyceride, cholesterol, apoB100, and apoB48 levels in ApoE^−/−^ mice fed on high-fat diet (Table 1). Moreover, in in vitro experiments, COS treatment increased the expression of *scavenger receptor BI* (*SR-BI*) and *ATP binding cassette transporter A1* (*ABCA1*) in macrophages, and *SR-BI* and *low-density lipoprotein receptor* (*LDL-R*) in the liver (Figure 1). Interestingly, the plasma lipid level was not affected in LDL-R-deficient mice, thus suggesting that the COS lipid-lowering effect is mostly based on the LDL-R pathway [30]. Later, these in vivo results were confirmed in another study, which also provided further elucidation of the underlying molecular mechanism in the HepG2 cells treated with COS. Interestingly, while the LDL-R and HMG-CoA reductase (HMGCR) protein levels were not affected, the mRNA levels of proprotein convertase subtilisin/kexin type 9 (PCSK9) were down-regulated by COS treatment. Further differences were observed between the cell lysates, with an increased expression of *Sterol-responsive element-binding protein 2* (*SREBP-2*) and hepatocyte nuclear factor-1α (HNF-1α) in the total cell lysates, while the levels of active nuclear subunit (nSREBP-2) were decreased, and the levels of forkhead box O3 (FOXO3a) were increased, in nuclear lysates after COS treatment. The combined in vivo and in vitro results suggested that COS acted on two transcription factors regulating PCSK9 synthesis at the gene transcription level, SREBPs and HNF-1α. Subsequently, PCSK9 (a known LDL-R inhibitor) modulated the hepatic LDL-R abundance and activity, thus improving the lipid intake by the liver, and decreasing the lipid concentration in the serum (Figure 1) [31].

Recently, lipid-lowering properties have also been demonstrated for cationically modified chitosan (via the covalent attachment of glycidyltrimethylammonium chloride), which inhibited atherosclerotic plaque development and modulated the expression of lipid metabolism genes. In particular, treatment with modified COS decreased the plasma LDL cholesterol level by 32%, and reduced the area of the atherosclerotic plaque by 33% in ApoE^−/−^ mice. Additionally, the expression level of *HMGCR* was reduced in HepG2 cells after incubation with modified chitosan [32]. Similarly, interesting results were demonstrated for chitosan–fucoidan nanoparticles (CFNs), which were able to bind with P-selectin, and exhibited antioxidant and anti-inflammatory activities. Furthermore, CFNs intravenously injected into atherosclerotic ApoE^−/−^ mice accumulated in atherosclerotic plaques, subsequently decreasing the average plaque area (by 36.5%), and the necrotic core area, and enhancing the fibrous cap thickness around the plaques, thus stabilising the atherosclerotic plaques. At the same time, long-term CFN treatment did not cause any significant adverse effects on the heart, liver, lungs, spleen, or kidneys, thus confirming its safety [33]. Together, the in vitro and in vivo results suggested that COS effectively reduced lipid levels, and suppressed local oxidative stress and inflammation, by targeting P-selectin in atheromatous plaques, and modulating the lipid metabolism, thereby preventing the progression of atherosclerosis.

## 4. Heparin-Derived Oligosaccharides

Heparin-derived oligosaccharides (HDOs) are delivered from heparin via enzymatic or chemical degradation, and have an ultra-low molecular weight and a narrow range of molecular weight distribution. HDOs have been proven to exhibit anti-proliferative and anti-inflammatory effects, which could be beneficial in the treatment of atherosclerosis [59,60]. As has been shown in rabbits fed a high-fat diet with vascular intimal hyperplasia in a balloon-injured carotid artery, HDOs could ameliorate the intimal hyperplasia, and inhibit the histopathology and restenosis induced by the balloon injury (Table 1). The HDO treatment decreased the expression of *SR-BI*, *MCP-1*, *VCAM-1*, *vascular endothelial growth factor* (*VEGF*), and *basic fibroblast growth factor* (*bFGF*) in the arterial wall. Additionally, the serum levels of lipids (total cholesterol, HDL, LDL, and triglycerides) were decreased. Interestingly, the expression level of *ABCA1* was increased (Figure 1) [34]. ABCA1 is the major regulator of cellular cholesterol homeostasis, and is closely associated with atherosclerosis development [61,62].

The proliferation and migration of the vascular smooth muscle cells (VSMCs) play a crucial role in atherosclerosis development. Many growth factors (such as VEGF, bFGF, and platelet-derived growth factor (PDGF) can act through protein kinase C (PKC), the protein kinase B/phosphoinositide 3-kinase (Akt/PI3K) pathway, or mitogen-activated protein kinase (MAPK), to promote VSMC proliferation [63]. HDOs have been demonstrated to inhibit the expression of VEGF receptors 1 and 2, and interrupt normal binding between VEGF and VEGF receptors, thus acting as a VEGF antagonist. Moreover, the expression of *MAPK*, *PI3K*/*Akt*, and *PKC* was inhibited, thus suggesting a high potential for the application of HDOs for atherosclerosis prevention and cure [35]. Therefore, HDOs affect a wide variety of metabolic pathways, which could be potentially used to treat atherosclerosis. However, the underlying molecular pathways require a detailed investigation in future experiments.

## 5. Galacto-Oligosaccharides

Galacto-oligosaccharides (GOSs) are β(1,4)- or β(1,6)-linked oligosaccharides made up of galactose, with glucose/galactose present at the terminal end. Despite their direct effect on the gut microbiota and host immunity, a number of studies suggest various other biological activities [64]. The effect of GOSs on the lipid profile is one of the best-studied aspects in rat models of different diseases. For example, GOS administration effectively decreased the levels of total cholesterol, LDL, and VLDL cholesterol, and triglycerides in hypercholesterolaemic rats fed on a high-fat diet (Table 1) [36]. Similarly, GOS supplementation ameliorated the metabolic alterations induced by a high-fat Western-type diet in Ldlr^−/−^ mice. In particular, lower levels of circulating macrophages and neutrophils (by 30 and 60%, respectively), suggested a reduced systemic inflammation. Moreover, GOS administration was associated with a reduced atherosclerotic lesion area, improved glucose tolerance, and reduced plasma lipopolysaccharide (LPS) concentrations [37]. Therefore, these results suggest that GOSs could be beneficial in the prevention and treatment of atherosclerosis and associated cardiovascular diseases.

Recently, GOSs were effectively used to improve the levels of HDL cholesterol, and reduce the levels of total cholesterol, triglycerides, and LDL cholesterol in the obesity rat model. GOSs also increased the expression levels of fat-browning genes (such as *PR domain containing 16* (*PRDM16*), *peroxisome proliferator-activated receptor-γ* (*PPARγ*), *peroxisome proliferator-activated receptor-γ coactivator 1α* (*PGC1α*), and *uncoupling protein 1* (*UCP1*)), thus promoting the browning of white fat cells, and the thermogenesis of brown fat cells. Furthermore, GOSs increased the levels of low-density lipoprotein receptor, peroxisome proliferation-activated receptor-α (PPARα), cholesterol 7α-hydroxylase proteins, and LXRα proteins in the liver of obese rats (Figure 1). Therefore, GOS inhibits obesity in rats, by facilitating white-fat browning and thermogenesis, and promoting cholesterol catabolism [38]. However, other, contradicting results were demonstrated in the rat model of streptozotocin-induced diabetes. GOS supplementation reduced the levels of MDA, total cholesterol, and CRP in diabetic rats, but had no effect on the concentrations of triglycerides and IL-6. Thus, GOSs showed a beneficial antioxidant property, while the effect on the inflammatory and lipid profiles was minor [39], suggesting that further studies are required, to establish and compare the detailed effects of GOSs in models of different diseases.

## 6. Cyclodextrins

Cyclodextrins (CDs) are cyclic oligosaccharides produced from various starch sources (such as corn or potatoes) by cyclodextrin glycosyltransferase, which can split and rearrange the polymer chain of starch to α-1,4-D-glucopyranoside oligomers. Common cyclodextrins are α-, β-, and γ-cyclodextrins, which are composed of six, seven, and eight glucose moieties, respectively [65]. The ability of cyclodextrins (especially β-cyclodextrin) to entrap cholesterol into their hydrophobic cavity and form stable inclusion complexes has been intensively exploited to extract excess cholesterol from atherosclerotic lesions in experimental animals [66,67]. Additionally, various physical and chemical methods have been used to modify cyclodextrins with the purpose of improving some of their characteristics (such as to improve solubility in water, decrease toxicity, and increase the ability to form complexes). At the same time, modifications take place in the hydroxyl groups (directed outwards) of cyclodextrins, thus the hydrophobicity and cavity diameter are not affected [68]. Later in this section, we focus on recent achievements in the application of various cyclodextrins in relation to atherosclerosis treatment. The application of CDs in pharmaceuticals as drug carriers has been covered in other reviews, and will be omitted here. We wish to redirect readers interested in this and related topics to the papers [69,70,71,72].

### 6.1. Effects on Lesion and Plaque Size and Cholesterol Transport

Experiments with ApoE^−/−^ mice fed on a high-fat diet and supplemented with α-CD and β-CD demonstrated a 65% decrease in aortic atherosclerotic lesions in the α-CD group, and a decrease in the intestinal lipid absorption and the plasma level of free fatty acids in the β-CD group, while the major plasma lipids and body weight parameters were not affected in either experimental group (Table 1) [40]. Further in vitro experiments on aortic bovine ECs and SMCs showed that methylated β-CD decreased cholesterol release and the expression levels of *ABCA1* and *ABCG1* transporters, which are crucial players in regulating the cellular cholesterol pools (also known as reverse cholesterol transport, or RCT) [41]. Later, these results were confirmed on rabbits fed a high-fat diet and supplemented with 2-hydroxypropyl-β-cyclodextrin (HPβCD), which reduced inflammatory cytokine and adhesion protein (TNF-a, IL-6, IL-8, MCP-1, VCAM-1, and ICAM-1) and plasma triglyceride levels, while increasing the plasma HDL cholesterol levels. Additionally, the atherosclerosis lesion area, and collagen and macrophage content in the lesions were reduced. Furthermore, the expression levels of the *ABCA1* and *ABCG1* transporters in the liver and aortic plaque were increased [43]. Another β-CD modification, cyclodextrin polymer (CDP), inhibited plaque growth in a mouse model of atherosclerosis more effectively, compared to HPβCD. Moreover, CDP demonstrated better pharmacokinetics and reduced ototoxicity at high doses, thus overcoming the known limitations of CD therapy for the treatment of atherosclerosis [42].

Furthermore, HPβCD treatment reduced the atherosclerosis plaque size and cholesterol crystal load, and facilitated plaque regression in ApoE^−/−^ mice on a continued cholesterol-rich diet. On a molecular level, HPβCD acted by targeting the liver X receptor (LXR) genes, thus reprogramming the cholesterol efflux (activating ABCA1 and ABCG1), and providing an anti-inflammatory effect (modulating the expression of the inflammasome sensor *NLRP3* (*NLR family pyrin domain containing 3*), and the pro-inflammatory cytokines *IL-1β* and *IL-18*). The in vivo application of HPβCD on patients led to an increase in cholesterol excretion into the urine, suggesting that HPβCD extracts and transports cholesterol directly, or promotes RCT from macrophages in an LXR-dependent way (Figure 1) [44]. Similarly, experiments on macrophages showed that HPβCD can extract free cholesterol from aggregated LDL, and reduce the cholesterol uptake and cholesteryl ester accumulation from aggregated LDL by macrophages [45].

However, recent research has not confirmed the lesion-regression effect of HPβCD in atherosclerotic ApoE^−/−^ mice fed on a high-fat diet. At the same time, experiments with macrophages have verified the ability of HPβCD to induce the cholesterol efflux from macrophages, thus supporting previous findings [73]. Therefore, despite the demonstrated safety, and beneficial effects on the key mechanisms of atherogenesis both in vitro and in vitro, further confirmatory studies are required on the regression-inducing potential of some cyclodextrin modification variants. The observed discrepancies in in vivo experiments suggest that the molecular mechanism of the HPβCD action still requires further investigation in order to be completely understood, and finely controlled, at the cellular level.

### 6.2. LDL Oxidation

Oxidised LDL (oxLDL) is one of the mmLDL forms implicated in atherosclerosis pathogenesis, and an important risk marker for human cardiovascular diseases [74]. It has been shown that α-CD, HPβCD, MβCD, and γ-CD can cause a significant separation between the lipid and protein components of native LDL. Furthermore, all four tested CDs (with the most pronounced results shown for HPβCD and MβCD) can reduce the LDL susceptibility to copper-induced oxidation in a concentration-dependent manner (Table 1) [46]. Similarly, MβCD and HPβCD have been shown to inhibit lipoxygenase-induced LDL oxidation in a concentration-dependent way, while increasing the lipoxygenase-induced oxidation of the lipids depleted from LDL [47]. However, it is necessary to note that the CDs were used at a relatively high concentration, and the results were demonstrated only in vitro. Therefore, further research is required to confirm these promising results in vivo.

### 6.3. Adhesion to Endothelial Cells

The adhesion of leukocytes to ECs promotes inflammation and endothelial dysfunction, and thus plays an important role in atherosclerosis pathogenesis. Multiple research studies have confirmed that cyclodextrins (mostly βCD and its modifications) can reduce leukocyte adhesion to ECs. As has been demonstrated, MβCD can impair the adhesion of THP-1 monocytes to HUVECs by decreasing the expression of *ICAM-1* and modulating membrane–cytoskeleton coupling. In particular, MβCD reduced the average length of the individual actin filaments, and reduced the expression of the *caveolin-1* and *Phosphatidylinositol 4,5-bisphosphate* (*PIP2*) levels, two major components of caveolae, and thus impaired the adhesion of circulating leukocytes to HUVECs, which could potentially help to inhibit inflammation and the initiation/progression of atherosclerosis [48].

**Figure 2:**
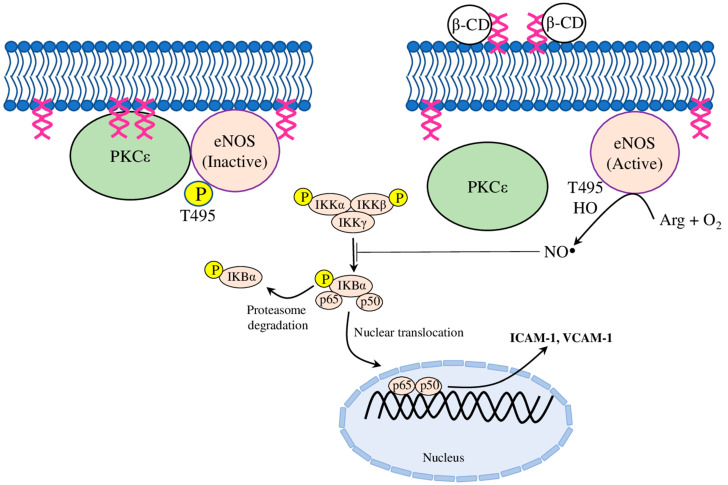
The proposed mechanism of β-cyclodextrin action. β-CD binds and forms a β-CD-DAG complex with diacylglycerol (DAG), which prevents the recruitment of PKCε (protein kinase Cε) to the plasma membrane by DAG, and thus inactivates PKCε. Furthermore, the blocked PKCε-catalysed phosphorylation of eNOS (endothelial nitric oxide synthetase) at the active site T-495 activated eNOS, which produced NO (nitric oxide), and inhibited the activation of the nuclear factor kappa B (NFκB) pathway. Therefore, β-CD-mediated NO production blocked the expression of *intercellular adhesion molecule-1* (*ICAM-1*) and *vascular cell adhesion molecule-1* (*VCAM-1*), thereby inhibiting monocytic adhesion to endothelial cells.

Recently, several molecular mechanisms of the βCD have been suggested. Firstly, βCD was found to regulate NO production in bovine aortic endothelial cells by inactivating the protein kinase Cε (PKCε), which is known to dephosphorylate T495 in endothelial nitric oxide synthetase (eNOS) (Figure 2). Furthermore, the elevated NO level inhibited IκB degradation, and the subsequent TNFα-induced nuclear factor kappa B (NFκB) activation, thus blocking *ICAM-1* and *VCAM-1* expression, and thereby inhibiting monocyte adhesion to ECs (Table 1) [49]. Secondly, another mechanism has been identified on oxLDL and LPS treated HUVECs, whereby MβCD reduced by 2–3 times the expression of *ICAM-1* and *VCAM-1*, acting through the IKK-NFκB (for LPS) or Akt-NFκB (for oxLDL) pathway [50]. Finally, luminol-conjugated β-cyclodextrin nanoparticles (LCD NP) inhibited neutrophil and macrophage infiltration, and subsequent pro-inflammatory events, in both in vitro and in vivo experiments. Accordingly, neutrophils treated with LCD NP reduced the phorbol 12-myristate 13-acetate (PMA)-induced production of TNFα, IL-1β, and ROS. The treatment of RAW264.7 macrophages with LCD NP inhibited their MCP-1-stimulated migration. Furthermore, long-term LCD NP administration in ApoE^−/−^ mice fed on a high-fat diet showed an accumulation of nanoparticles in atherosclerotic plaques, which was associated with reduced plaque formation, a reduced neutrophil and macrophage number, and an increased collagen content around plaques [51]. Together, these results proposed potential mechanisms for the anti-atherosclerotic effects of CDs from the angle of monocyte–endothelial adhesion and plaque stabilisation.

### 6.4. Complement Activation

The complement system is a crucial part of innate immunity, which is known to contribute to the pathology of various inflammatory diseases. There are three known pathways to activate the complement system: the classical pathway, the lectin pathway, and the alternative pathway [75]. The classical pathway is activated by the direct binding of the pattern-recognition molecule C1q to various structures and ligands on pathogens or apoptotic cells, or indirectly via binding to other molecules, such as immunoglobulins or C-reactive protein. The lectin pathway is initiated through soluble mannose-binding lectin (MBL) and ficolins, which can recognise a wide variety of microorganisms, altered self-structures, and acetylated compounds, respectively [76]. The alternative pathway is activated via the spontaneous hydrolysis of the internal C3 thioester in the fluid phase, or directly on foreign surfaces [77]. Additionally, the alternative pathway functions to substantially amplify the activation induced by the classical and lectin pathways. All three pathways rely on the central C3 molecule, leading to the cleavage of C3 into its active fragments C3a and C3b, and the subsequent cleavage of C5 to C5a and C5b, and the further assembly of the terminal complement complex. Moreover, C3a and C5a are potent anaphylatoxins that can induce both sterile and nonsterile inflammatory processes [78].

It is known that cholesterol crystals have employed the complement system to activate cytokine production. Acting through both the classical and alternative complement pathways, cholesterol crystals induced the release of cytokines (IL1-β and TNFα) and ROS production, and up-regulated the production of active caspase-1 and complement receptor 3 (or CD18), with the latter leading to the phagocytosis of cholesterol crystals [79]. Further investigation showed that cholesterol crystals and complement initiated plaque inflammation, by activating the NLRP3 inflammasome. Accordingly, the complement C1q and C5b-9 complex accumulated around cholesterol crystal clefts, and the complement receptors *C5aR1*, *C5aR2*, and *C3aR1* were higher in the carotid plaques of acute coronary syndrome patients. Furthermore, the priming of human carotid plaques with C5a, and incubation with cholesterol crystals increase the release of IL-1α, IL-1β, and IL-18; and priming with C5a and TNFα, and incubation with cholesterol crystals up-regulated the plaque expression of *NLRP3* inflammasome components [80]. Similarly, cholesterol crystals activated the lectin pathway by binding with MBL and ficolin-2 (in a calcium-dependent and calcium-independent way, respectively). Furthermore, IgM was bound to cholesterol crystal, and facilitated its binding with C1q in vitro [81].

Recent research demonstrated that CDs were effective inhibitors of the cholesterol crystal-mediated activation of the complement system. It was shown that HPβCD inhibited C3c binding to cholesterol crystals, and reduced the terminal complement complex (TCC) generation and deposition on the surface of cholesterol crystals in human plasma, by reducing C1q and ficolin-2, and Igs (IgM and IgA) deposition on the surface of cholesterol crystals. Interestingly, HPβCD application reduced the expression of *CR1* and *CR3* receptors in monocytes, but not in granulocytes, where the phagocytosis of cholesterol crystals was increased (Table 1). However, the cholesterol crystal-stimulated ROS production was decreased after HPβCD treatment in both monocytes and granulocytes. Furthermore, the expression of key cytokines (such as *TNFα*, *IL-1α*, *IL-1β*, *IL-6*, and *IL-8*), NLRP3-related genes, and macrophage inflammatory protein-1α (MIP-1α) was decreased after exposure to HPβCD [52]. Later investigation has defined similar properties for αCD, which also inhibited cholesterol crystal-induced complement activation (by preventing the binding of C1q and ficolin-2), reduced ROS production in both monocytes and granulocytes, and reduced phagocytosis. Interestingly, unlike HPβCD, αCD could dissolve cholesterol crystals [53]. Together, these results suggested CDs as a potential treatment to prevent cholesterol crystal-induced complement activation and inflammation in atherosclerosis.

## 7. Oligosaccharides Which Stimulate Atherosclerosis

The association between the galactose-α-1,3-galactose (α-Gal) oligosaccharide and atherosclerosis was recently demonstrated. The novel α-Gal syndrome was described as an IgE-mediated immediate-type allergy to α-Gal [82]. The total IgE and α-Gal specific IgE were tested in the sera of 118 patients who had been subjected to medically warranted cardiac catheterisation and who had undergone an intravascular ultrasound. It was shown that α-Gal-specific IgE to α-Gal was detected in 26% of cases, and the atheroma burden and unstable features of the plaques were higher in sensitised subjects. The most pronounced association between the IgE to α-Gal and the atherosclerosis burden was defined in elder subjects (≤65 years of age) [83]. A further cross-sectional study among patients with, or at risk of, coronary artery disease (CAD) identified an association between α-Gal sensitisation and CAD burden and ST-segment-elevation myocardial infarction (STEMI). Independently of other risk factors, α-Gal sensitisation was associated with the presence of non-calcified plaque and obstructive CAD. Furthermore, the α-Gal sensitisation rate was higher in patients with STEMI than in the patients with CAD [84], thus proposing IgE sensitisation to α-Gal as a novel risk factor for coronary atherosclerosis.

Interestingly, a common disaccharide sucrose was recently identified as the main driver of the metabolic inflammation promoting atherosclerosis in LDLr^−/−^ ApoB^100/100^ hyperlipidaemic mice. Mice fed on a high-fat/low-sucrose diet were characterised by pronounced dyslipidaemia, insulin resistance, and obesity, compared to mice fed on a low-fat/high-sucrose diet. On the other hand, a high-sucrose diet was associated with a reduced macrophage cholesterol efflux capacity, higher adipose tissue inflammation, liver inflammation and fibrosis, ~80% enhanced aortic atherosclerosis area, and left ventricular enlargement [85]. However, further studies, especially in human patients, are required, to define the exact impact of these results, because they could provide new insights into the pathogenesis of atherosclerosis, other cardiovascular diseases, and liver diseases.

## 8. Conclusions

**Figure 3:**
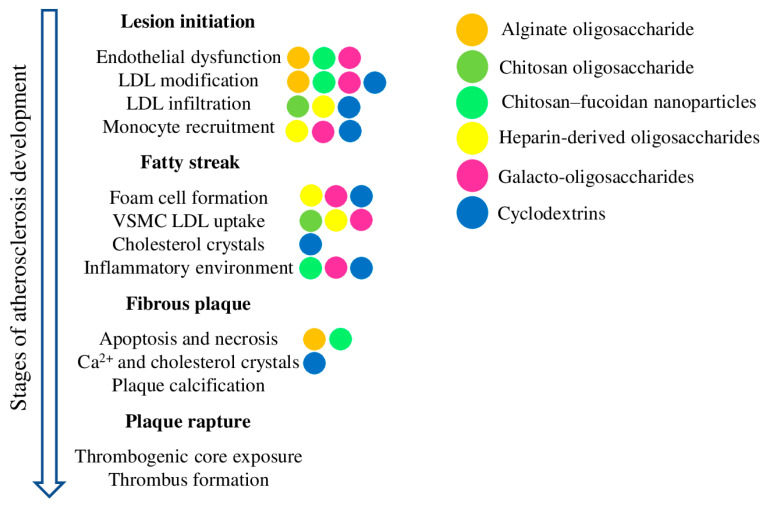
Schematic representation of atherosclerosis development and the most important processes contributing to each stage. The coloured dots depict the effects of the corresponding oligosaccharides on the given process.

Oligosaccharides derived from natural sources have many practical aspects that make them appealing therapeutics for the chronic processes associated with atherosclerosis. Despite a centuries-long history of consumption by domestic animals and humans supporting the safety of oligosaccharides in a diet, the exact molecular mechanisms of their action are mostly unknown, and require further research. The discussed oligosaccharides have demonstrated encouraging results in terms of increased antioxidant defence, reduced atherosclerotic plaque size, inhibited cholesterol uptake and foam cell formation, and arrested cholesterol crystal-induced complement activation and inflammation progress (Figure 3). Furthermore, modern methods of enzymatic degradation, polymerisation, chemical modification, and nanomodification can help to improve the pharmacokinetics and bio-distribution of oligosaccharides, thus improving anti-atherosclerotic activities while reducing side effects. Overall, there is great therapeutic potential in oligosaccharides, with the added benefits of a low cost, low toxicity, and abundant supply, enabling their chronic dietary administration to prevent and treat atherosclerosis, diabetes, obesity, and related diseases.

## Figures and Tables

**Table 1:** The anti-atherosclerotic effects of the discussed oligosaccharides.

Oligosaccharide	General Effect of Oligosaccharide Treatment	Proposed Molecular Mechanism	Reference
Alginate oligosaccharide (AOS)—2 to 25 monomers of β-1,4-mannuronic acid and α-1,4-guluronic acid boned by 1, 4-glycosidic linkages	increased mouse survival rate and improved DOX-induced cardiac dysfunction, and attenuated myocardial apoptosis	decreased the expression of pro-apoptotic proteins (*CHOP* and *Bax*) and increased the expression of anti-apoptotic protein *Bcl-2*	[24]
	decreased myocardial apoptosis and attenuated oxidative stress; decreased the infarct size and ameliorated cardiac dysfunction after I/R injury in mice	decreased generation of superoxide, 4-hydroxynonenal, and 3-nitrotyrosine; down-regulated NADPH oxidase 2 and inducible nitric oxide synthase; up-regulated Blc-2, and down-regulated CHOP, caspase-12, Bax, and glucose-regulated protein 78	[26]
	protected HUVECs against oxidative stress-induced apoptosis	regulated the expression of *P21*, *FAK*, *CDK2*, *integrin-α*, *PTEN*, and *PI3K;* increased the expression of *Bcl-2*, and decreased levels of caspase 3 and Bax	[27]
	protected HUVECs against H_2_O_2_-induced oxidative stress and apoptosis	up-regulated antioxidant enzymes (SOD and CAT) and glutathione, and reduced the activities of caspase-3 and caspase-9	[28]
	preserved ejection fraction and fractional shortening; improved mitochondrial biogenesis, turn-over and integrity in mouse model of D-galactose-induced cardiac ageing	inhibited the D-galactose-induced up-regulation of ageing markers p21 and p53, and cardiac peptide hormones BNP and ANP; increased the mtDNA copy number, autophagy rate, sustained mitochondrial membrane potential and up-regulated the expression of *PGC-1α* and *SIRT3*	[29]
Chitosan oligosaccharide (COS), a linear oligomer composed of β-(1 ➔ 4)-linked N-acetyl-d-glucosamine and d-glucosamine	decreased the lesion and plaque areas in aortic root, increased plaque stability, and reduced plasma triglycerides, cholesterol, apoB100, and apoB48 levels in ApoE^−/−^ mice fed on HFD	increased the expression of *SR-BI* and *ABCA1* in macrophages in vitro, and *SR-BI* and *LDL-R* in liver	[30]
	improved lipid uptake by liver and decreased lipid concentration in the serum	down-regulated the mRNA levels of PCSK9; increased the expression of *SREBP-2* and HNF-1α in total cell lysates; decreased the levels of active nSREBP-2, and increased the levels of FOXO3a in nuclear lysates	[31]
	demonstrated lipid-lowering properties, inhibited atherosclerotic plaque development	decreased the plasma LDL cholesterol level by 32%; reduced the area of atherosclerotic plaque by 33% in ApoE^–/–^ mice; reduced the expression of *HMGCR* in HepG2 cells	[32]
chitosan–fucoidan nanoparticles (CFNs)	exhibited antioxidant and anti-inflammatory activities; intravenously injected CFNs decreased the average plaque area (by 36.5%), and necrotic core area, and enhanced the fibrous cap thickness around the plaques, thus stabilising atherosclerotic plaques in atherosclerotic ApoE^–/–^ mice	-	[33]
Heparin-derived oligosaccharides (HDOs); these are structurally diverse, ranging in size from disaccharide to tetradecasaccharide	ameliorated intimal hyperplasia and inhibited the histopathology and restenosis induced by balloon injury in rabbits fed a high-fat diet	decreased expression of *SR-BI*, *MCP-1*, *VCAM-1*, *VEGF*, and *bFGF* in the arterial wall; decreased the serum levels of total cholesterol, HDL, LDL, and triglycerides; increased the expression level of *ABCA1*	[34]
	acted like VEGF antagonist in culture of human aortic smooth muscle cells	inhibited the expression of VEGF receptors 1 and 2, and interrupted normal binding between VEGF and VEGF receptors; inhibited the expression of *MAPK*, *PI3K*/*Akt*, and *PKC*	[35]
Galacto-oligosaccharides (GOS); these consist of β-linked galactose moieties with galactose or glucose at the reducing end, different degrees of polymerisation and configurations	demonstrated lipid-lowering properties in hypercholesterolaemic rats fed on high-fat diet,	decreased levels of total cholesterol, LDL and VLDL cholesterol, and triglycerides	[36]
	reduced atherosclerotic lesion area; ameliorated metabolic alterations induced by a high-fat Western-type diet in Ldlr^−/−^ mice	decreased levels of circulating macrophages and neutrophils (by 30 and 60%, respectively); improved glucose tolerance and reduced plasma LPS concentrations	[37]
	facilitated white-fat browning and thermogenesis, and promoted cholesterol catabolism, thus inhibiting obesity in rats	improved levels of HDL cholesterol and reduced levels of total cholesterol, triglycerides, and LDL cholesterol; increased the expression of fat-browning genes (*PRDM16*, *PPARγ*, *PGC1α*, and *UCP1*); increased levels of LDL-R, PPARα, cholesterol 7α-hydroxylase proteins and LXRα proteins	[38]
	demonstrated antioxidant, lipid-lowering, and anti-inflammatory properties in a rat model of streptozotocin-induced diabetes	reduced levels of MDA, total cholesterol, and CRP	[39]
α- and β- Cyclodextrins (α-CD and β-CD), comprising glucose molecules in the pyranose (six-membered) ring configuration; 6, 8, or 10 glucopyranosides bind with each other to form α-, β-, and γ-cyclodextrin, respectively.	α-CD decreased the aortic atherosclerotic lesions by 65% in ApoE^−/−^ mice fed on high-fat diet	β-CD decreased the intestinal lipid absorption and plasma level of free fatty acids	[40]
β-CD	regulated the cellular cholesterol pools in vitro in aortic bovine ECs and SMCs	decreased cholesterol release and the expression levels of *ABCA1* and *ABCG1* transporters	[41]
cyclodextrin polymer (CDP)	inhibited plaque growth in a mouse model of atherosclerosis; demonstrated better pharmacokinetics and reduced ototoxicity at high doses	-	[42]
2-hydroxypropyl-β-cyclodextrin (HPβCD)	reduced the atherosclerosis lesion area and collagen and macrophage content in the lesions in rabbits fed a high-fat diet	reduced inflammatory cytokine and adhesion proteins (TNF-a, IL-6, IL-8, MCP-1, VCAM-1, and ICAM-1) and plasma triglyceride levels; increased plasma HDL cholesterol levels; increased the expression of *ABCA1* and *ABCG1* transporters in livers and aortic plaques	[43]
	reduced atherosclerosis plaque size and cholesterol crystal load, and facilitated plaque regression in ApoE^−/−^ mice on a continued cholesterol-rich diet	activated ABCA1 and ABCG1, modulated the expression of the inflammasome sensor *NLRP3* and pro-inflammatory cytokines (*IL-1β* and *IL-18*); increased cholesterol excretion into the urine in human patients	[44]
	extracted free cholesterol from aggregated LDL in macrophages	reduced cholesterol uptake and cholesteryl ester accumulation from aggregated LDL	[45]
α-CD, HPβCD, methyl-βCD (MβCD), and γ-CD	caused a significant separation between lipid and protein components of native LDL in vitro	reduced LDL susceptibility to copper-induced oxidation in a concentration-dependent manner	[46]
methyl-βCD and HPβCD	demonstrated anti-oxidant properties in vitro	inhibited lipoxygenase-induced LDL oxidation in a concentration-dependent way, increased the lipoxygenase-induced oxidation of the lipids depleted from LDL	[47]
MβCD	impaired the adhesion of THP-1 monocytes to HUVECs	decreased the expression of *ICAM-1* and modulated membrane-cytoskeleton coupling; reduced the average length of individual actin filaments and reduced the expression of *caveolin-1* and *PIP2* levels	[48]
βCD	regulated NO production in bovine aortic ECs; inhibited monocyte adhesion to ECs	inactivated PKCε, inhibited IκB degradation and subsequent TNFα-induced NFκB activation, thus blocking *ICAM-1* and *VCAM-1* expression	[49]
MβCD	suppressed LPS/oxLDL-induced monocyte-HUVEC adhesion	reduced by 2–3 times the expression of *ICAM-1* and *VCAM-1*	[50]
luminol-conjugated β-cyclodextrin nanoparticles (LCD NP)	inhibited neutrophil and macrophage infiltration and subsequent pro-inflammatory events in both in vitro and in vivo experiments; long-term administration resulted in NP accumulation in atherosclerotic plaques, reduced plaque formation, reduced neutrophils and macrophages number, and increased collagen content around plaques in ApoE^−/−^ mice fed on a high-fat diet	reduced phorbol 12-myristate 13-acetate-induced production of TNFα, IL-1β, and ROS in neutrophils inhibited the MCP-1-stimulated migration of RAW264.7 macrophages	[51]
HPβCD	inhibited C3c binding to cholesterol crystals, reduced TCC generation and deposition on the surface of cholesterol crystals through reduced C1q and ficolin-2, and Igs (IgM and IgA) deposition on the surface of cholesterol crystals; increased the phagocytosis of cholesterol crystals in granulocytes	reduced the expression of *CR1* and *CR3* receptors in monocytes decreased cholesterol crystal-stimulated ROS production in both monocytes and granulocytes; decreased expression of key cytokines (such as *TNFα*, *IL-1α*, *IL-1β*, *IL-6* and *IL-8*), NLRP3 related genes and MIP-1α	[52]
αCD	inhibited cholesterol crystal-induced complement activation; reduced phagocytosis in monocytes and granulocytes	prevented binding of C1q and ficolin-2, reduced ROS production in monocytes and granulocytes	[53]

## Data Availability

Not applicable.
